# Monitoring Avian Influenza Viruses from Chicken Carcasses Sold at Markets, China, 2016

**DOI:** 10.3201/eid2310.170679

**Published:** 2017-10

**Authors:** Xiaoxiao Mao, Jie Wu, Eric H.Y. Lau, Kit Ling Cheng, Zhifeng Zhong, Yinchao Song, Xunmin Ji, Lirong Zhou, Changwen Ke, Joseph Sriyal Malik Peiris, Hong Wang, Hui-Ling Yen

**Affiliations:** South China Agriculture University, Guangzhou, China (X. Mao, H. Wang);; Guangdong Provincial Center for Disease Control and Prevention, Guangzhou (X. Mao, J. Wu, Z. Zhong, Y. Song, L. Zhou, C. Ke);; The University of Hong Kong, Hong Kong, China (E.H.Y. Lau, K.L. Cheng, J.S.M. Peiris, H.-L. Yen)

**Keywords:** avian influenza, dressed poultry, chickens, viral load, food safety, markets, viruses, China, zoonoses, poultry, influenza

## Abstract

During 2016 in Guangzhou, China, we detected infectious avian influenza viruses (AIVs) in 39.8% of samples from chicken carcasses slaughtered at live poultry markets but none from carcasses supplied to supermarkets by facilities bypassing live poultry markets. Promoting supply chains with high biosecurity may reduce the risk for zoonotic AIV transmission.

Live poultry markets (LPMs) are hot spots for avian influenza virus (AIV) amplification among poultry and dissemination to humans (*1*). Direct contact with live poultry is a major route of zoonotic transmission (*2*), but field data are limited on risks from contaminated poultry carcasses (*3*). Control measures, including market rest-days and ban of live poultry stalls in urban areas with central slaughtering and sales of poultry carcasses, have been implemented in China (*3**,**4*). In the city of Guangzhou (population 14 million), 200,000 live poultry (*5*) and ≈81,000 freshly processed poultry carcasses are supplied daily. Live poultry is supplied from 6 wholesale LPMs to ≈600 retail LPMs (*5*). Poultry carcasses are supplied through 2 sources: 1) poultry sourced from multiple independent poultry farms and slaughtered at the wholesale LPMs or 2) private poultry slaughtering industries with integrated supply chains that bypass the LPM system (*6*).

Low pathogenic AIV replicates in the respiratory and gastrointestinal epithelium cells of infected birds; highly pathogenic AIV can replicate systematically in multiple tissues (*7*). In avian species, the lungs where AIV can replicate are entrenched into the ribs and cannot be removed during poultry slaughtering; furthermore, AIV has been detected in air sacs from experimentally inoculated chickens (*8*). The mass slaughtering process also provides an opportunity for cross-contamination through common use of tools or water. Previous studies have reported detection of infectious highly pathogenic AIV (H5N1, H5N2, H5N3, and H7N1) and low pathogenic AIV (H9N2) subtypes from poultry meat after natural infections or after experimental inoculations (*8**–**14*). However, how frequently infectious AIV can be detected from processed poultry carcasses is unclear. We report the detection of viral RNA and infectious AIV from freshly processed chicken carcasses sold at different markets in Guangzhou, China.

## The Study

During June–November 2016, we sampled fresh chicken carcasses supplied from the LPM system. Samples were collected twice each month from dressed poultry stalls within 1 wholesale LPM and from 3 retail LPMs located in different districts in Guangzhou. Dressed poultry or poultry carcasses are prepared similarly as in other countries (e.g., defeathered and eviscerated); however, the head and feet remain with the carcass. A total of 1,230 swabs were collected from the oropharynx, cloacal cavity, and visceral cavity of chicken carcasses supplied from the LPM system (Table 1).

**Table 1:** AIV detected from chicken carcasses sold at live poultry markets, dressed poultry stalls, or supermarkets, Guangzhou, Guangdong Province, China, June–December 2016*

Swab type	No. qRT-PCR positive/no. tested (%)					No. culture positive/no. tested (%)†			
Dressed poultry stall‡	Retail market§	Supermarket¶	p value#	Dressed poultry stall‡	Retail market§	Supermarket¶	p value#
Oropharyngeal	67/121 (55.4)	207/277 (74.7)	2/62 (3.2)	0.097		44/121 36.4)	158/277 (57.0)	0/62 (0)	0.026
Cloacal	55/120 (45.8)	177/265 (66.8)	4/62 (6.5)	0.053		38/120 31.7)	133/265 (50.2)	0/62 (0)	0.033
Visceral cavity	48/118 (40.7)	203/329 (61.7)	2/23 (8.7)	0.033		23/118 19.5)	93/329 (28.3)	0/23 (0)	0.151

*AIV, avian influenza virus; qRT-PCR, quantitative real-time reverse transcription PCR. †All qRT-PCR–positive samples were tested for infectivity in MDCK cells, and all qRT-PCR–negative samples were assumed negative for AIV infectivity. ‡Chickens centrally slaughtered at the wholesale market are sold at designated dressed poultry stalls without live poultry on site. §Data from 3 retail markets located at different districts in Guangzhou city. ¶Data from 3 supermarket chains with poultry supplied by different industrial suppliers. #Comparison of AIV-positive rates between dressed poultry stalls and retail markets by Fisher exact test.

During July–December 2016, we also sampled chilled chicken carcasses supplied from the private poultry slaughtering industries that bypass the LPM system. Chicken carcasses were sampled from 3 different supermarket chains once each month; 147 swabs were collected in virus transport media (Table 1). The quantity of AIV viral RNA segment 7 (matrix gene) was determined by quantitative real-time reverse transcription PCR (qRT-PCR) (*15*). and the infectious virus dose was determined by titration in MDCK cells, which are not as sensitive for AIV as the embryonated chicken eggs and might underestimate the rate of positivity. Influenza A virus matrix gene–positive samples were subtyped using H5-, H7-, or H9-specific primers and probes by qRT-PCR (*15*); the design of the primers and probes cannot differentiate whether the multiple basic amino acids are present at the hemagglutinin cleavage site.

The AIV-positive rates detected from fresh chicken carcasses varied by market type (Table 1). Rates of positive viral RNA detected from oropharyngeal, cloacal, or visceral cavity samples of chicken carcasses sold at the retail markets were 20% higher than were those collected from the dressed poultry stalls (all p<0.1 by Fisher exact test) and >50% higher than those collected from the supermarkets (all p<0.002 by Fisher exact test) (Table 1). Samples collected from retail markets had 9%–20% higher culture-positive rates than those collected from dressed poultry stalls (all p<0.2 by Fisher exact test); no culture-positive samples were identified from the supermarkets. qRT-PCR yielded higher rates of positivity than did the cell culture–based assay (all p<0.05 by Fisher exact test), possibly because of the inactivation of AIV during the scalding process, when the poultry carcasses are immersed in hot water (50°–64°C) for 45–120 s to loosen the feathers.

We detected significantly higher viral loads in oropharyngeal swabs than in cloacal or visceral cavity swabs of chicken carcasses sold at the dressed poultry stalls or the retail markets (Figure). Most AIVs detected belonged to H9 subtype, which is consistent with our previous report (*15*). We found samples positive for H7 or for H7 and H9 subtypes at a frequency of up to 6.3% (oropharyngeal swabs at the retail markets) (Table 2). We detected more diverse hemagglutinin subtypes from the chicken carcasses sold at the retail market than sold elsewhere; specifically, we detected H5 subtype only at retail market B.

**Figure:**
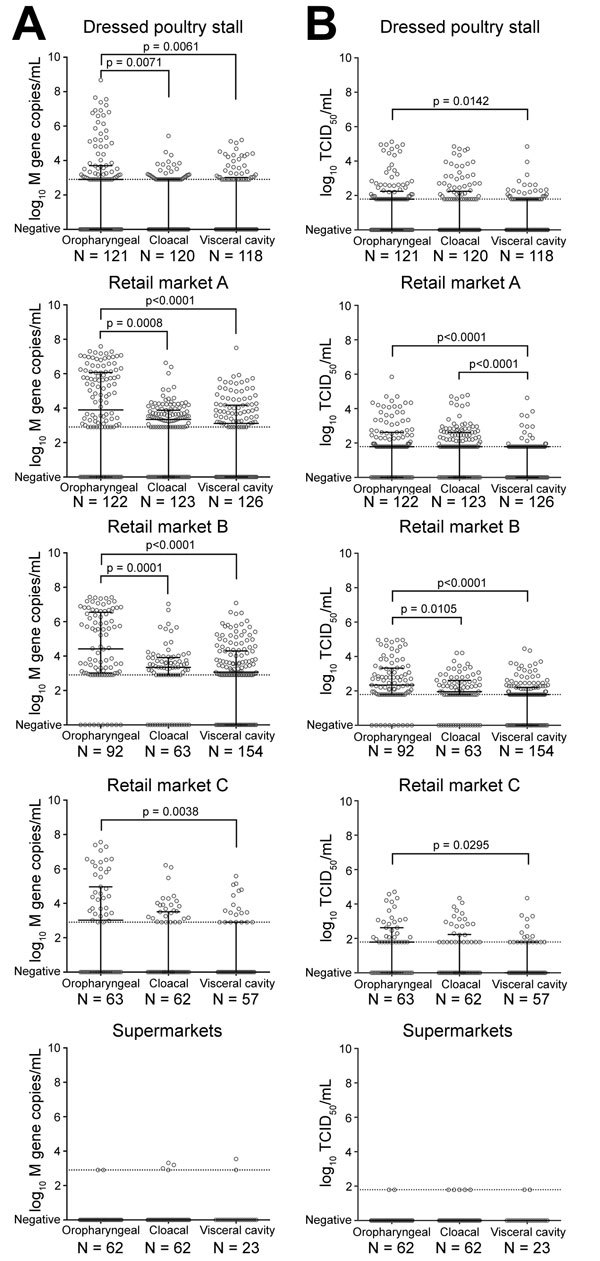
Copies of avian influenza virus RNA (A) and infectious viral loads (B) detected from chicken carcasses sold at live poultry markets or supplied through slaughtering industries, Guangzhou, Guangdong, China, June–December 2016. The median value with interquartile range is shown for each dataset. Dotted lines represent the limit of linear range of quantification for viral RNA (2.903 log_10_ matrix gene copies/mL) (A) or the detection limit by TCID_50_ assay (1.789 log_10_ TCID_50_/mL) in MDCK cells (B). p values from Kruskal-Wallis test followed by Dunn’s multiple comparison test are shown. AIV, avian influenza virus; TCID_50_, 50% tissue culture infectious dose.

**Table 2:** AIV subtypes detected by qRT-PCR from swabs of chicken carcasses sold at live poultry markets and from chilled poultry supplied through slaughtering industries, Guangzhou, Guangdong Province, China, June–December 2016*

Subtype	Wholesale market				Retail markets				Supermarket		
OP	Cloacal	Visceral cavity	OP	Cloacal	Visceral cavity	OP	Cloacal	Visceral cavity
H9	58/67 (86.6)	39/55 (70.9)	32/48 (66.7)		166/207 (80.2)	143/177 (80.8)	131/203 (64.5)		0/2	1/4 (25.0)	0/2
H5	0/67	0/55	0/48		2/207 (1.0)	1/177 (0.6)	4/203 (2.0)		0/2	0/4	0/2
H7	0/67	0/55	2/48 (4.2)		0/207	0/177	2/203 (1.0)		0/2	0/4	0/2
H5+H9	0/67	0/55	0/48		4/207 (1.9)	6/177 (3.4)	5/203 (2.5)		0/2	0/4	0/2
H7+H9	2/67 (3.0)	3/55 (5.5)	2/48 (4.2)		13/207 (6.3)	6/177 (3.4)	10/203 (5.0)		0/2	0/4	0/2
H5+H7+H9	0/67	0/55	0/48		3/207 (1.5)	5/177 (2.8)	7/203 (3.5)		0/2	0/4	0/2
Non-H5/H7/H9	4/67 (6.0)	3/55 (5.5)	1/48 (2.1)		10/207 (4.8)	7/177 (4.0)	27/203 (13.3)		0/2	1/4 (25.0)	1/2 (50.0)
Low copy (nontypeable)†	3/67 (4.5)	10/55 (18.2)	11/48 (22.9)		9/207 (4.4)	9/177 (5.1)	17/203 (8.4)		2/2 (100.0)	2/4 (50.0)	1/2 (50.0)

*Values are no. positive/no. tested (%). AIV, avian influenza virus; OP, oropharyngeal; qRT-PCR, quantitative real-time reverse transcription PCR. †Samples with matrix gene copy below linear range of quantification (2.903 log_10_ matrix gene copies/mL) and are negative for H5/H7/H9 by qRT-PCR.

## Conclusions

Our results agree with results from a previous study that reported detection of AIV RNA from chicken carcasses sold at retail and dressed poultry stalls in Guangzhou (*3*). However, the previous study did not provide data on virus viability. Our data demonstrate high levels (39.8% of 1,230 samples collected from carcasses) of contaminated chicken carcasses with infectious AIV supplied through the LPM system, either at the retail LPM or at the dressed poultry stalls. These results suggest potential infection risk for consumers through handling the poultry meat, contaminating other foods in the kitchen, or eating partially cooked poultry products. In contrast, we found no infectious AIV in the 147 chicken carcasses collected from supermarkets supplied through the integrated poultry production and slaughtering industries that bypass the LPM system. Chilling should not affect the sensitivity of qRT-PCR and might help sustain survival of infectious virus. Although we did not assess the potential difference in the slaughtering process at the private slaughtering industry and at LPMs, we believe qRT-PCR might still be sensitive enough to detect AIV-contaminated carcasses after extensive cleaning. Collectively, our results suggest that AIV amplification through poultry mixing and extended overnight stay within the wholesale or retail LPM system (*1*) might have contributed to contamination of carcasses.

In a separate study conducted during June–November 2016, we detected similar rates of positive viral RNA from the oropharyngeal swabs (172 [47.8%] of 360) (p = 0.172 by Fisher exact test) from live poultry sold at the same wholesale market as that detected from the chicken carcasses at the dressed poultry stalls (67 [55.4%] of 121) (Table 1). The result further supports that the AIV prevalence in the source poultry determines the level of residual AIV found on chicken carcasses.

In conclusion, our data suggest that chicken carcasses may pose a substantial zoonotic risk for AIV infection even in the absence of direct contact with live poultry. Central slaughtering might not by itself eliminate zoonotic risk if the source poultry have high rates of virus carriage. The LPM system in China continues to provide venues and opportunities of poultry mixing from different sources that facilitate AIV persistence and amplification despite interventions, such as market rest-days and banning the holding of live poultry overnight, that aim to reduce such a risk (*1*). In this regard, promoting vertically integrated supply chains of farms and slaughterhouses with high biosecurity would be a promising effective control measure to reduce the risk for zoonotic transmission of AIV.
